# Transgelin Inhibits the Malignant Progression of Esophageal Squamous Cell Carcinomas by Regulating Epithelial–Mesenchymal Transition

**DOI:** 10.3389/fonc.2021.709486

**Published:** 2021-08-26

**Authors:** Boli Yang, Qiuyu Chen, Changshan Wan, Siyuan Sun, Lanping Zhu, Zhizhong Zhao, Weilong Zhong, Bangmao Wang

**Affiliations:** ^1^ Department of Gastroenterology and Hepatology, Tianjin Medical University General Hospital, Tianjin Institute of Digestive Disease, Tianjin, China; ^2^ Department of Digestive Diseases, Jincheng General Hospital, Shanxi, China

**Keywords:** esophageal squamous cell carcinoma (ESCC), transgelin, epithelial mesenchymal transition (EMT), invasion, metastasis, proliferation

## Abstract

**Objective:**

This article investigates the role of Transgelin (TAGLN) in the epithelial–mesenchymal transition (EMT) of esophageal squamous cell carcinomas (ESCC) and its possible mechanism of inhibiting the invasion of these cancers.

**Methods:**

Tissue specimens and clinical information of patients with ESCC were collected to analyze the relationship between Transgelin expression level and prognosis of patients with ESCC. Transgelin siRNA was used to knock down Transgelin expression. The expression of Transgelin in Eca-109 and KYSE-150 cells was overexpressed by Transgelin-overexpressing plasmid. The effects of Transgelin overexpression and knockdown on the proliferation of Eca-109 and KYSE-150 cells were examined by Transwell chamber, scratch assay, and CCK-8 cell activity assay. RT-PCR and Western blot were used to detect the effect of Transgelin overexpression or knockdown on the mRNA and protein expressions of E-cadherin and Vimentin. TCGA data were used to analyze Transgelin co-expressed genes and further study the GO and KEGG enrichment analysis results under the influence of Transgelin.

**Results:**

The expression of Transgelin was low in ESCC, and its expression level was positively correlated with the prognosis of patients with ESCC. The targeted Transgelin siRNA and Transgelin-overexpressing plasmid can effectively regulate the expression of Transgelin mRNA and protein in Eca-109 and KYSE-150 cells. After overexpression of Transgelin, the invasion and proliferation abilities of Eca-109 and KYSE-150 cells were significantly decreased compared with those of the control group (P < 0.05). However, Transgelin knockdown could promote the proliferation, migration, and invasion of ESCC cells. The overexpression of Transgelin inhibits EMT in ESCC. With the increase of Transgelin expression in Eca-109 and KYSE-150 cells, the expression of E-cadherin increased, while the expression of Vimentin decreased, and the difference was statistically significant (P < 0.05).

**Conclusion:**

Transgelin can inhibit the malignant progression of ESCC by inhibiting the occurrence of EMT.

## Introduction

Esophageal squamous cell carcinoma (ESCC) is a common malignant tumor of the digestive tract with poor prognosis and high mortality (1, 2). ESCC is the 5th most common malignancy in men and the 8th most common malignancy in women (3). The incidence of ESCC has declined in recent years because of the advances in early cancer screening (4). However, ESCC treatment remains a difficult problem (5). At present, the most effective treatment for ESCC is surgery, chemotherapy, and radiotherapy as adjuvant treatment. There are abundant lymphocytes under the mucosa of the esophagus, so when the tumor encroaches below the mucosa of the esophagus, cancer cells can easily spread along these lymphocytes. In addition, the esophagus does not have a serous layer, which is less resistant to tumor invasion. When the tumor invades the muscle layer of the esophagus, it may invade the surrounding organs. There are more important organs near the esophagus. When the tumor grows and extends forward or backward, it is more difficult to remove it surgically or to treat it with radiation. Thus, finding more targeted therapeutic targets through in-depth research on the pathogenesis of ESCC is an urgent problem to be solved in clinical practice (6–8). In addition, the molecular mechanisms related to the pathogenesis and invasion of ESCC remain to be further explored (9).

Tumor invasion and metastasis are the main cause of death in cancer patients (10). Therefore, the mechanism of tumor invasion and metastasis is one of the hot issues in current research (11). Epithelial–mesenchymal transition (EMT) is one of the major factors in tumor cell invasion and metastasis (12). EMT is mainly characterized by the deletion of epidermal phenotype and acquisition of interstitial phenotype (13). EMT can not only enhance the proliferation and migration abilities of tumor cells, but also interact with the tumor microenvironment. EMT induces local tissue remodeling and imparts stem cell properties on tumor cells. Therefore, inhibiting the occurrence of EMT may be a new direction of tumor therapy (14).

Transgelin is a 22 kDa protein that is widely found in smooth muscle tissues (15). Transgelin is an actin gelatin that is sensitive to changes in transformation, mutation, and shape (16). However, few studies have investigated its function. Studies have shown that Transgelin gene expression is reduced or deleted in various tumors. In addition, some studies have found that Transgelin can also be used as signal molecules to participate in cell growth and extracellular matrix degradation and is expressed in various tumor tissues to varying degrees. Transgelin is closely related to the occurrence, development, and invasion of tumors (17–19). Recent studies have also shown that Transgelin plays an important role in tumor evolution (20). However, few study have investigated the mechanism of Transgelin involvement in ESCC invasion and metastasis through EMT.

The purpose of this study was to observe the effect of Transgelin on the invasion, metastasis, and proliferation of ESCC cell lines (Eca-109 and KYSE-150) by regulating their expression of Transgelin. Meanwhile, the effects of Transgelin on the expression of E-cadherin and Vimentin were observed to investigate the role and molecular mechanism of Transgelin in the occurrence, invasion, and metastasis of EMT in ESCC. This study aims to provide new ideas and effective approaches for the diagnosis and treatment of ESCC and to find new potential therapeutic targets.

## Methods

### Collection and Information of Clinical Samples

The pathological specimens of 74 ESCC patients were obtained from Tianjin Medical University General Hospital. Each specimen included esophageal carcinoma and adjacent normal tissue (distance from cancer tissue >5 cm). None of the patients received chemotherapy, radiotherapy, or other related antitumor therapies before surgery. The final follow-up time was December 31, 2020. In this study, The pathological types of LGIN, HGIN, and esophageal squamous cell carcinoma in the present study were determined by independent diagnosis by two pathologists (21, 22). The tumor stage and grade classification were based on the 8th American Joint Committee on Cancer (AJCC). This research was in accordance with the Helsinki Declaration of the World Medical Association. Every patient signed an informed consent form. The experiment was approved by the Ethics Committee of Tianjin Medical University General Hospital. The clinicopathological characteristics of patients are shown in **Table 1**.

**Table 1:** Clinical data were analyzed to investigate the relationship between the expression of Transgelin and pathological characteristics.

Parameters	Group	TAGLN expression (n=74)			*p*-value
		Cases	Negative	Positive	
Gender	Male	69	34	35	0.643
	Female	5	3	2	
Age (year)	<65	42	27	15	0.005**
	≥65	32	10	22	
T grade	T1 +T2	12	2	10	0.012*
	T3 +T4	62	35	27	
Lymphatic invasion	N0	31	10	21	0.01**
	N1-N3	43	27	16	
Differentiation	High + Moderate	37	18	19	0.816
	Low	37	19	18	
AJCC Stage	I-II	29	5	24	<0.001***
	III-IV	45	32	13	
Size (cm)	<3	36	15	21	0.163
	≥3	38	22	16	
Tumor site	Upper and median	42	22	20	0.639
	Lower	32	15	17	

P ＜ 0.05 represents statistical significance (chi-square test). *P < 0.05, **P < 0.01, and ***P < 0.001. Bold values: p < 0.05.

### Cell Culture

Human ESCC cell lines (Eca-109 and KYSE-150) were purchased from the Cell Bank of the Institute of Biochemistry and Cell Biology, Shanghai Institutes for Biological Sciences, Chinese Academy of Sciences. Eca-109 and KYSE-150 cells were cultured in DMEM containing 10% fetal bovine serum (Gibco, Life Technologies, Rockville, MD, USA) and incubated at 37°C in a 5% carbon dioxide incubator at constant temperature.

### Cell Transfection

Eca-109 and KYSE-150 cells were seeded into 6-well plates at 5 × 10^5^ cells/well for transfection. The culture density was 50%–60%. According to the instructions of Lipofectamine 2000 (Life Technologies, Rockville, MD, USA), two different siRNAs against Transgelin and corresponding negative control siRNAs were transfected into Eca-109 and KYSE-150 cells. The final transfection concentration was 100 nmol/L. After 6 h of culture in serum-free medium, the culture was transferred to 10% serum medium for 24 h. Cells were collected for subsequent experiments. Transgelin-overexpressing Transgelin plasmids (Transgelin cDNA ORF Clone, Human, pCMV3-N-HA as carrier, SinoBiological, China) and no-load control plasmids were transfected with the same method. The final transfection concentration was 1mg/L. The experimental cells were divided into the following groups: Vector-NC (empty plasmid), Transgelin (Transgelin-overexpressing plasmid), si-NC (negative control siRNA), and si-Transgelin (Transgelin siRNAs). All cell phenotype experiments are completed within 48 hours after the completion of transfection.

### Detection of Cell Activity by CCK-8 Assay

The transfected cells in the growth phase were taken. Cells were seeded into 96-well plates with 5000 cells per well. After conventional culture for 24, 48, and 72 h, 10 μL of CCK-8 solutions (Beyotime, Shanghai, China) was added to each well. After incubation at 37°C for 1 h, the absorbance (A) value of each well at 450 nm was detected with a microplate analyzer (Multiskan EX, Lab systems, Helsinki, Finland), and the cell viability was calculated.

### Cell Scratch Test

Before the wound scratch, incubate the cells with serum-free medium overnight to inhibit cell proliferation on migration. After digestion, the cells were collected and seeded into 6-well plates with 2 × 10^5^ cells/well for culture. When the fusion degree of cell culture reached 100%, the cell center was scratched linearly. Next, 1× phosphate buffer solution (PBS) was used to wash the cell suspension. Serum-free DMEM (Life Technologies) was added to each well. The cells were cultured at 37°C in a cell incubator under 95% relative humidity and 5% CO_2_. Changes of cell migration were observed at 0, 12, 24, and 36 h. Each experiment was repeated three times.

### Transwell Invasion Test

Matrigel (BD, Biosciences, Franklin Lakes, NJ, USA) was dissolved overnight at 4°C and diluted in complete medium (1:3). About 50 μL of the mixture was added to the upper and lower compartment of each 24-well plate (Millipore, Billerica, MA, USA). Then, the samples were placed into a 37°C cell incubator and set for 30 min to coagulate. Logarithmic growth cells were digested and collected. About 2 × 10^4^ cells were washed in 200 μL of serum-free medium. Resuspended cells were added to the upper compartment. About 600 μL of complete medium was added to the lower chamber. The cell culture plates were cultured at 37°C in an incubator under 95% relative humidity and 5% CO_2_. After 24 h, Matrigel and cells in the upper compartment were removed. The submembrane compartment surface was stained with crystal violet and photographed for cell counting. Nikon (Japan) frontal microscope was used to randomly select 10 fields to count the number of cells, and the average value was obtained. Each experiment was repeated three times.

### Detection of the Migration Ability of ESCC Cells in Each Group by Transwell Migration Assay

Unlike the invasion assay, the Transwell migration assay did not require Matrigel-embedded chambers for 1 h before cell inoculation. The remaining steps are the same as those in the Transwell invasion experiment.

### QRT-PCR

Cells in each group were collected, and total RNA was extracted by Trizol method. cDNA was synthesized using a reverse transcription kit (Takara, Japan). The amplification was performed using a real-time fluorescent quantitative PCR apparatus in accordance with the instructions of the SYBR Green Kit (Invitrogen, Carlsbad, CA, USA). PCR was used to amplify Transgelin, E-cadherin, Vimentin, and GAPDH. The sequence was as follows: Transgelin upstream primer: 5′-GGTGGAGTGGATCATAGTGC-3′, downstream primer: 5′-ATGTCAGTCTTGATGACCCCA-3′; E-cadherin upstream primer: 5′-GACGCG GACGATGATGTGAAC-3′, downstream primer: 5′-TTGTACGTGGTGGGATTG AAGA-3′; Vimentin upstream primer: 5′-GACAATGCGTCTCTGGCACGTCTT-3′, downstream primer: 5′-TCCTCCGCCTCCTGCAGGTTCTT-3′; internal reference upstream primer: 5′-CCCTTCATTGACCTCAACTACATGG-3′, downstream primer: 5′-CATGGTGGTGAAGACGCCAG-3′. The reaction conditions were as follows: 95°C for 30 s; 95°C for 8 s, 60°C for 32 s, 40 cycles; 95°C for 1 min, 60°C for 30 s, 95°C for 30 s. The 2^-ΔΔCT^ method was used to calculate the relative gene expression. A single experiment was repeated for three times, and three duplicate wells were set in each experiment.

### Western Blot

The total cell protein was extracted and quantified by BCA method. The sample quantity was 40 μg. The sample was separated by 10% SDS-PAGE. The membrane was transferred to the PVDF membrane by using the wet method (1 kDa/min), and the primary antibody was added after 1 h with TBST sealant containing 5% skim milk powder. Primary antibody was added and incubated overnight at 4°C. Anti-TAGLN/Transgelin (ab233971), abcam; Vimentin (D21H3) XP^®^ Rabbit mAb #5741, CST; E-Cadherin (24E10) Rabbit mAb #3195, CST; N-Cadherin (D4R1H) XP^®^ Rabbit mAb #13116, CST; Claudin-1 (D5H1D) XP^®^ Rabbit mAb #13255, CST; Rabbit anti-Catenin-gamma Polyclonal Antibody, abs131596, absin; Mouse Anti-β actin mAb, TA-09 ZSGB-BIO, Beijing, China; Mouse Anti-GAPDH mAb, TA-08 ZSGB-BIO, Beijing, China. The dilution ratio of antibody was 1:1000. The secondary antibody (Horseradish enzyme labeled goat anti-rabbit IgG (H+L), ZB-2301, ZSGB-BIO; Horseradish enzyme labeled goat anti-mouse IgG (H+L) ZB-2305, ZSGB-BIO) was added after washing the membrane with TBST and incubated at room temperature for 1 h. ECL glowed after washing the TBST film. The semi-quantitative analysis of the absorbance of the strips was carried out with ImageJ software. The ratio of the absorbance value of the target protein band to the absorbance value of the internal reference GAPDH and β-actin protein band was used to represent the relative amount of the target protein.

### Immunohistochemical Staining

All specimens were fixed in 10% neutral formaldehyde. Paraffin embedded, 4 μm continuous section. Xylene dewaxing, gradient ethanol hydration. Antigen thermal repair with 0.01 mol/L sodium citrate buffer. Endogenous peroxidase was sealed at 37°C for 20 min. Primary antibody (Transgelin, 1:200, Abcam; CD31, 1:1000, Abcam) was added and incubated overnight at 4°C. The secondary antibody was added and incubated at 37°C for 30 min. Horseradish peroxidase was added and incubated at 37°C for 20 min. DAB staining, hematoxylin redyeing, gradient ethanol dehydration. Xylene transparent, neutral gum sealed sheet, light microscope detection and photography. The staining results were graded according to the staining intensity and percentage of positive cells. The percentages of positive cells <5%, 5%–25%, 26%–50%, 51%–75%, and >75% were 0, 1, 2, 3, and 4, respectively. The cell staining intensity was scored as follows: 0 for non-staining, 1 for light yellow, 2 for brownish yellow, and 3 for yellowish brown. The positive intensity is the product of two scores: 0–2 is negative, 3–5 is weakly positive, 6–8 is positive, and 9–12 is strongly positive. The results were determined by two pathologists, and the average value was taken.

### CancerSEA Analysis

CancerSEA (http://biocc.hrbmu.edu.cn/CancerSEA/home.jsp) is a comprehensive database that provides the single-cell functional states of cancer cells at different sites. In this study, we investigated the correlation between Transgelin gene and different functional states of tumors and different cancer types through this website. The average correlation between Transgelin and functional status in different cancers, including invasion, differentiation, metastasis, cell cycle, stemness, and proliferation, was analyzed through this website.

### Transgelin Survival Analysis

The data were constructed using the Kaplan–Meier Plotter (https://kmplot.com/analysis/) database based on the GEO, EGA, and TCGA public database of gene chip and RNA-seq. The effects of 54,675 genes on survival in 21 types of cancer were assessed. The Kaplan–Meier Plotter database integrates gene expression information and clinical prognostic value for meta-analysis and survival-related molecular marker research, discovery, and validation. This website was used to analyze the prognostic value of Transgelin in different tumor types. In the analysis, the Kaplan–Meier Plotter database divided patients into two groups on the basis of different quantiles of Transgelin expression. The Kaplan–Meier survival chart was used to compare two cohorts and calculate the HR, 95% CI, and log rank P values.

### Protein–Protein Interaction (PPI) Network Analysis

The study of the interaction network between proteins is helpful to mine the core regulatory genes. In this study, STRING (https://string-db.org/) was used to analyze the PPI network relationship of Transgelin interacting proteins. The STRING database searches for known and predicted interactions between proteins. The database can be applied to 2031 species, containing 9.6 million proteins and 13.8 million protein interactions. In addition to experimental data, mined results from PubMed abstracts, and integrated data from other databases, the STRING database also contains the predicted results using bioinformatics methods.

### LinkedOmics Analysis

In this study, the LinkedOmics database was used to analyze the co-expressed genes of Transgelin and their participation in GO and KEGG enrichment analysis. Login LinkedOmics Database (http://linkedomics.org/login.php), the first to register to obtain access to the database. Then, screening conditions were selected successively in the database retrieval interface, and data related to Transgelin gene expression level and prognosis in ESCC were downloaded.

### Statistical Analysis

GraphPad Prism 7 software (GraphPad Software, Inc., San Diego, CA, USA) was used for statistical analysis. The measurement data were expressed as mean ± standard deviation. Two-tailed unpaired *t*-test was used for the comparison between two groups. One-way ANOVA was used for the comparison between multiple groups, and Tukey’s test was used for further pairwise comparison. The Pearson correlation coefficient was used for correlation analysis. The Kaplan–Meier method was used to calculate the relationship between Transgelin expression and survival prognosis of tumor patients. Statistical significance was considered at P < 0.05.

## Results

### Expression of Transgelin Was Low in ESCC, and Its Expression Level Was Positively Correlated With the Prognosis of ESCC

**Figure 1:**
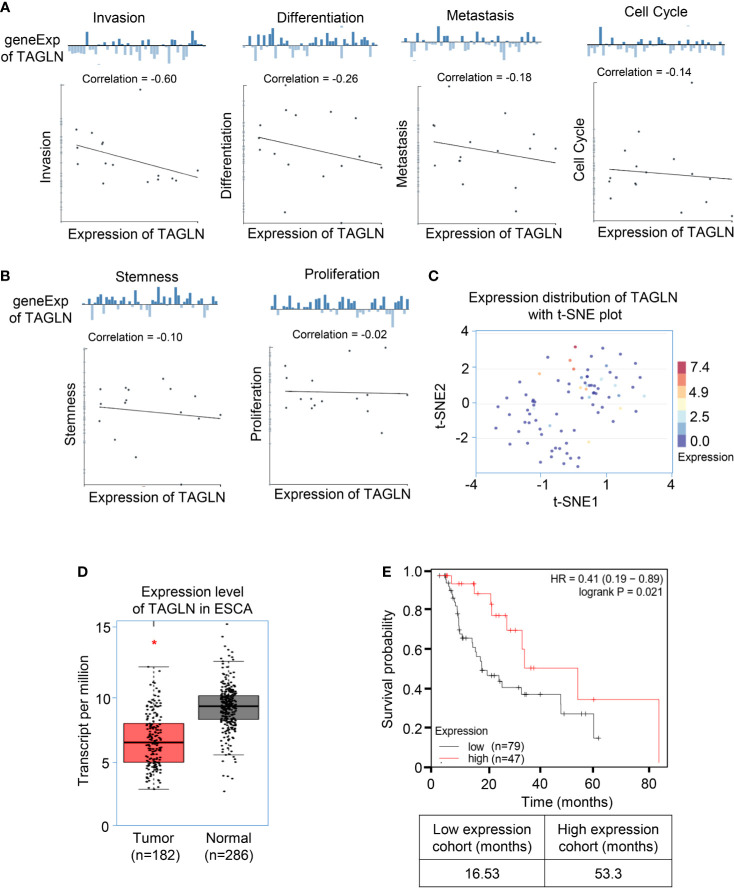
The expression of Transgelin was low in esophageal squamous cell carcinomas, and its expression level was positively correlated with the prognosis of esophageal squamous cell carcinomas. **(A)** The average correlation between Transgelin and functional status of ESCC. The analysis results show that the expression level of Transgelin is negatively correlated with Invasion, Differentiation, Metastasis and Cell Cycle. **(B)** The expression level of Transgelin is negatively correlated with Stemness and Proliferation. **(C)** The distribution of Transgelin expression in tumors and the t-SNE diagram of all individual cells. **(D)** Expression level of Transgelin in ESCC. **(E)** The correlation between Transgelin expression and prognosis in patients with esophageal squamous cell carcinomas. *P < 0.05.

We analyzed the average correlation between Transgelin and functional status, including invasion, differentiation, metastasis, cell cycle, stemness, and proliferation, in different cancers through the CancerSEA database. The analysis results show that the expression level of Transgelin is negatively correlated with invasion, differentiation, metastasis, cell cycle, stemness, and proliferation (**Figures 1A, B**). These results indicated that the high expression of Transgelin could reduce the invasion and metastasis of tumors. Furthermore, we analyzed the distribution of Transgelin expression in tumors and the t-SNE diagram of all individual cells, in which the color represented the expression level of the input gene. The analytical results showed that cells with low Transgelin expression were clustered together (**Figure 1C**). The expression results of Transgelin in ESCC and paracancerous tissues in the GEPIA Database (http://gepia.cancer-pku.cn/index.html) are shown in **Figure 1D**. The results showed that the expression level of Transgelin in ESCC tissues was lower than that in normal esophageal tissues in the dataset of ESCC collected by TCGA (P < 0.05). Through the above database mining information, we found that the expression of Transgelin was decreased in ESCC tissues. To further clarify the relationship between Transgelin expression and prognosis of ESCC, we analyzed the correlation between Transgelin expression and prognosis in patients with ESCC in the Kaplan–Meier Plotter database. Kaplan–Meier analysis showed that the expression level of Transgelin gene was correlated with the survival prognosis of ESCC patients. Patients with high Transgelin expression had a lower overall mortality, while patients with low Transgelin expression had poorer prognosis, and the difference was statistically significant (**Figure 1E**, P < 0.05). Prognostic analysis of Transgelin in breast cancer, liver hepatocellular carcinoma, and sarcoma also showed that patients with high Transgelin expression had a long survival time (**Supplementary Figure 1**).

### Overexpression of Transgelin Inhibits the Proliferation, Migration, and Invasion of ESCC Cells

**Figure 2:**
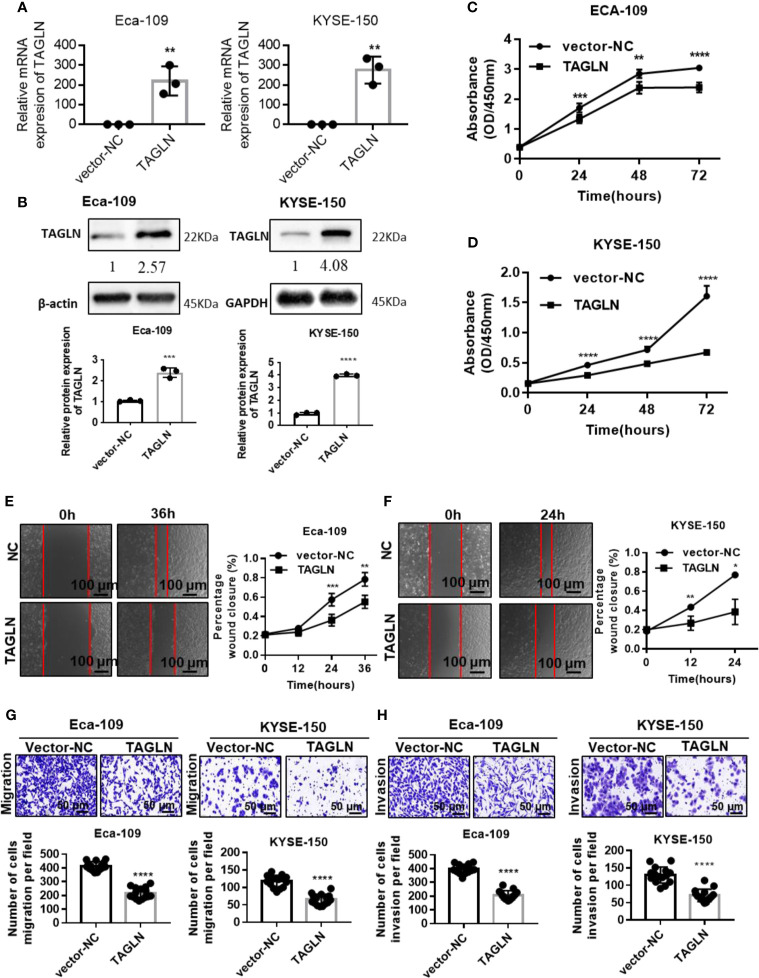
Over-expression Transgelin suppresses the proliferation, migration and invasive abilities of esophageal squamous cell carcinomas cells *in vitro*. **(A)** Eca-109 and KYSE-150 cells transfected with Transgelin overexpression vector, respectively. The expression of Transgelin was verified by qRT-PCR. **(B)** Eca-109 and KYSE-150 cells transfected with Transgelin overexpression vector, respectively. The expression of Transgelin was verified by western blotting. GAPDH and β-Actin were used as internal controls. **(C)** The effects of Transgelin overexpression on the proliferation ability was analyzed by CCK-8 kit in Eca-109 cells. **(D)** The effects of Transgelin overexpression on the proliferation ability was analyzed by CCK-8 kit in KYSE-150 cells. **(E)** The effects of Transgelin overexpression on the migration ability was analyzed by Scratch test in Eca-109 cells. **(F)** The effects of Transgelin overexpression on the migration ability was analyzed by Scratch test in KYSE-150 cells. **(G)** The effects of Transgelin overexpression on the migration ability was analyzed by transwell assay in Eca-109 and KYSE-150 cells. **(H)** The effects of Transgelin overexpression on the invasion ability was analyzed by transwell assay in Eca-109 and KYSE-150 cells The data are presented as the mean ± SEM of three independent experiments. *P < 0.05, **P < 0.01, ***P < 0.001, and ****P < 0.0001 by two-tailed Student’s t-test.

The qRT-PCR results showed that Transgelin mRNA expression level in Eca-109 and KYSE-150 cells transfected with Transgelin-overexpressing plasmid was significantly higher than that in the empty plasmid group, and the difference was statistically significant (**Figure 2A**, P < 0.01). Conforming to the qRT-PCR results, the Western blot results showed that Transgelin protein expression levels in Eca-109 and KYSE-150 cells transfected with Transgelin-overexpressing plasmids were also significantly higher than those in the control group, and the difference was statistically significant (**Figure 2B**, P < 0.01). The results indicated that the Transgelin plasmid used in the experiment could effectively promote the expression of Transgelin *in vitro*. The CCK-8 results showed that Transgelin overexpression in Eca-109 and KYSE-150 cells significantly reduced the proliferation capacity of cells, and the difference was statistically significant (**Figures 2C, D**, P < 0.05). With the extension of transfection time, the inhibitory effect of Transgelin overexpression on the proliferation of Eca-109 and KYSE-150 cells increased continuously, and the difference was statistically significant (P < 0.05). These results indicated that the overexpression of Transgelin gene could effectively inhibit the proliferation ability of Eca-109 and KYSE-150 cells *in vitro*. The scratch test results showed that after Transgelin overexpression of Eca-109 and KYSE-150 cells, the migration ability of the overexpression group was significantly decreased compared with that of the blank control group, and the difference was statistically significant (**Figures 2E, F**, P < 0.05). Transgelin experiment results showed that after Transgelin overexpression in Eca-109 and KYSE-150 cells, the number of transmembrane-penetrating cells decreased significantly compared with the blank control group (**Figures 2G, H**, P < 0.05). These results indicated that the promotion of Transgelin expression could effectively inhibit the invasion and migration of Eca-109 and KYSE-150 cells *in vitro*.

### Effects of Transgelin Overexpression on mRNA and Protein Expression of E-Cadherin, Claudin-1, N-Cadherin, γ-Catenin and Vimentin in Eca-109 and KYSE-150 Cells

**Figure 3:**
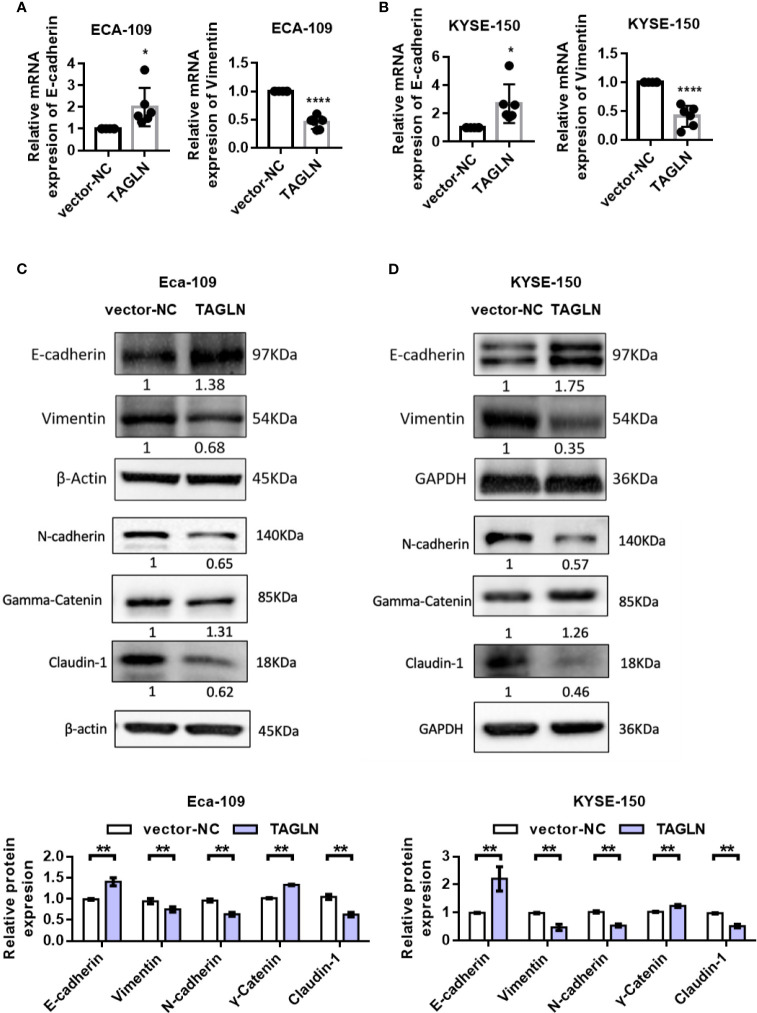
Effects of overexpression of Transgelin on E-cadherin and Vimentin expression in Eca-109 and KYSE-150 cells. **(A)** The expression levels of E-cadherin and Vimentin in Eca-109 cells after Transgelin overexpression were detected by qRT-PCR. **(B)** The expression levels of E-cadherin and Vimentin in KYSE-150 cells after Transgelin overexpression were detected by qRT-PCR. **(C)** The expression levels of E-cadherin, Claudin-1, N-cadherin, γ-Catenin and Vimentin in Eca-109 cells after Transgelin overexpression were detected by western blot. **(D)** The expression levels of E-cadherin, Claudin-1, N-cadherin, γ-Catenin and Vimentin in KYSE-150 cells after Transgelin overexpression were detected by western blot. The data are presented as the mean ± SEM of three independent experiments. *P < 0.05, **P < 0.01, and ****P < 0.0001 by two-tailed Student’s t-test.

The RT-PCR results showed that the expression of E-cadherin in Eca-109 cells was significantly increased with the increase of E-cadherin expression. However, the expression level of Vimentin was significantly decreased (**Figure 3A**). The results in KYSE-150 cells were consistent with those in Eca-109 (**Figure 3B**). Furthermore, the protein levels of E-cadherin, Claudin-1, N-cadherin, γ-Catenin and Vimentin after Transgelin overexpression were detected by Western blot. The experimental results showed that after Transgelin overexpression in Eca-109 and KYSE-150 cells, the content of E-cadherin and γ-Catenin protein in the cells increased, whereas the expression of Vimentin, N-cadherin and Claudin-1 protein in the cells decreased accordingly; the difference was statistically significant (**Figures 3C, D**, P < 0.05). These results indicated that Transgelin could inhibit EMT by regulating the expression of epithelial phenotype and mesenchymal phenotype in Eca-109 and KYSE 150 cells.

### Effects of Transgelin Knockdown on the Proliferation, Migration, and Invasion of Eca-109 and KYSE-150 Cells

**Figure 4:**
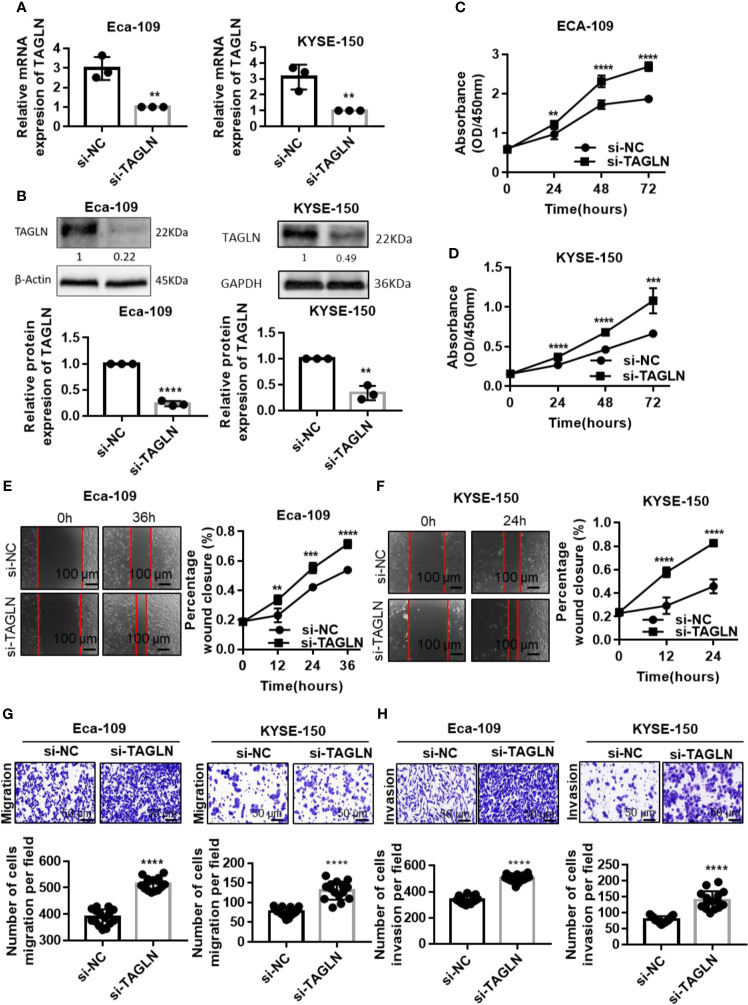
Inhibition of Transgelin promoted the proliferation, migration and invasive abilities of esophageal squamous cell carcinomas cells *in vitro*. **(A)** Eca-109 and KYSE-150 cells transfected with siRNA to silence Transgelin, respectively. The expression of Transgelin was verified by qRT-PCR. **(B)** Eca-109 and KYSE-150 cells transfected with siRNA to silence Transgelin, respectively. The expression of Transgelin was verified by western blotting. GAPDH and β-Actin were used as internal controls. **(C)** The effects of knockdown of Transgelin on the proliferation ability was analyzed by CCK-8 kit in Eca-109 cells. **(D)** The effects of knockdown of Transgelin on the proliferation ability was analyzed by CCK-8 kit in KYSE-150 cells. **(E)** The effects of knockdown of Transgelin on the migration ability was analyzed by Scratch test in Eca-109 cells. **(F)** The effects of knockdown of Transgelin on the migration ability was analyzed by Scratch test in KYSE-150 cells. **(G)** The effects of knockdown of Transgelin on the migration ability was analyzed by transwell assay in Eca-109 and KYSE-150 cells. **(H)** The effects of knockdown of Transgelin on the invasion ability was analyzed by transwell assay in Eca-109 and KYSE-150 cells. The data are presented as the mean ± SEM of three independent experiments. **P < 0.01, ***P < 0.001 and ****P < 0.0001 by two-tailed Student’s t-test.

Transgelin mRNA and protein expression levels in Eca-109 and KYSE-150 cells transfected with siRNA were significantly lower than those in the negative control group, and the difference was statistically significant (**Figures 4A, B**, P < 0.01). This finding indicated that the Transgelin siRNA used in the experiment could effectively inhibit the expression of Transgelin gene *in vitro*. The CCK-8 results showed that the cell proliferation rate was significantly enhanced after transfection of Transgelin siRNA in Eca-109 and KYSE-150 cells, and the difference was statistically significant (**Figures 4C, D**, P < 0.05). Moreover, with the extension of transfection time, the promotion of Transgelin siRNA on the proliferation of Eca-109 and KYSE-150 cells was enhanced. These results indicated that inhibiting Transgelin gene expression could effectively promote the proliferation ability of Eca-109 and KYSE-150 cells *in vitro*. The scratch test results showed that after Eca-109 and KYSE-150 cells were transfected with Transgelin siRNA, the migration ability of the interference group was significantly enhanced compared with that of the negative control group, and the difference was statistically significant (**Figures 4E, F**, P < 0.05). The transwell experiment results showed that Transgelin siRNA transfection in Eca-109 and KYSE-150 cells significantly increased the number of transmembrane cells (**Figures 4G, H**, P < 0.05). These results indicated that inhibiting Transgelin gene expression could effectively promote the invasion abilities of Eca-109 and KYSE-150 cells *in vitro*. To avoid the off-targets effects, another siRNAs against Transgelin was also used and completed related experiments, including cell transfection, qRT-PCR, Western Blot, CCK-8 assay, Cell scratch test, and Transwell assay. The results have been added to the **Supplementary Figure 3**. Those results showed that inhibit of Transgelin promoted the proliferation, migration, and invasive abilities of esophageal squamous cell carcinomas cells *in vitro*.

### Transgelin Knockdown Promoted EMT of ESCC

**Figure 5:**
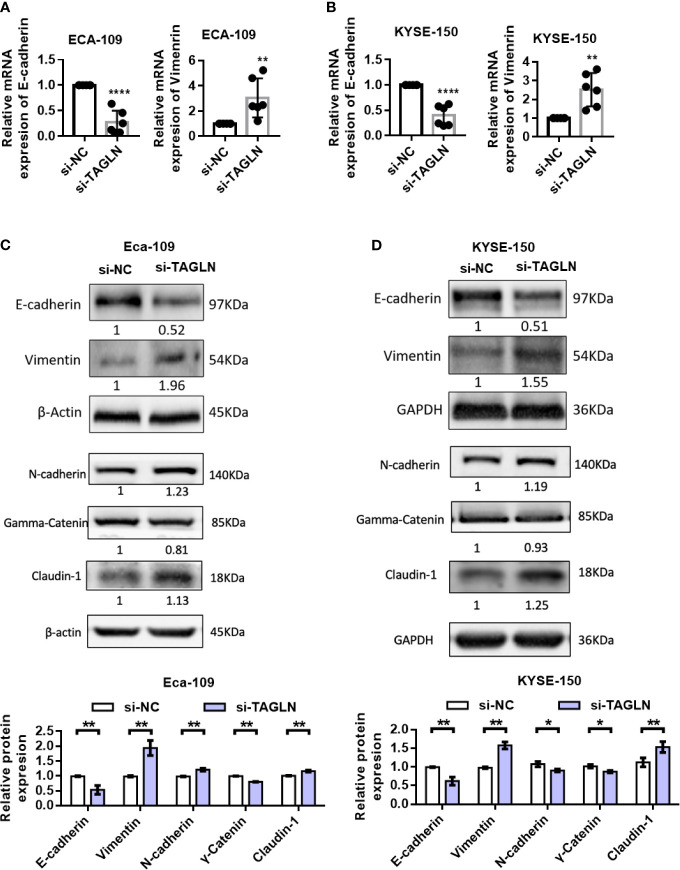
Effects of knockdown of Transgelin on E-cadherin and Vimentin expression in Eca-109 and KYSE-150 cells. **(A)** The expression levels of E-cadherin and Vimentin in Eca-109 cells after knockdown of Transgelin were detected by qRT-PCR. **(B)** The expression levels of E-cadherin and Vimentin in KYSE-150 cells after knockdown of Transgelin were detected by qRT-PCR. **(C)** The expression levels of E-cadherin, Claudin-1, N-cadherin, γ-Catenin and Vimentin in Eca-109 cells after knockdown of Transgelin were detected by western blot. **(D)** The expression levels of E-cadherin, Claudin-1, N-cadherin, γ-Catenin and Vimentin in KYSE-150 cells after knockdown of Transgelin were detected by western blot. The data are presented as the mean ± SEM of three independent experiments. *P < 0.05, **P < 0.01, and ****P < 0.0001 by two-tailed Student’s t-test.

First, we used RT-PCR to detect the changes in the expression levels of E-cadherin and Vimentin after Transgelin knockdown. The results showed that Transgelin inhibition significantly reduced the expression of E-cadherin in Eca-109 and KYSE-150 cells. By contrast, the expression level of Vimentin was significantly increased (**Figures 5A, B**). Furthermore, the protein levels of E-cadherin, Claudin-1, N-cadherin, γ-Catenin and Vimentin after Transgelin overexpression were detected by Western blot. The results showed that Transgelin knockdown in Eca-109 and KYSE-150 cells reduced the expression of E-cadherin and γ-Catenin protein. However, the expression of Vimentin, N-cadherin and Claudin-1 proteins in cells was upregulated, and the difference was statistically significant (**Figures 5C, D**, P < 0.05). This finding suggested that Transgelin knockdown promoted EMT in ESCC. The same results were obtained using si-TAGLN-2 to knock out TAGLN (**Figure S4**).

### Expression of Transgelin Decreased Gradually With the Progression of ESCC

**Figure 6:**
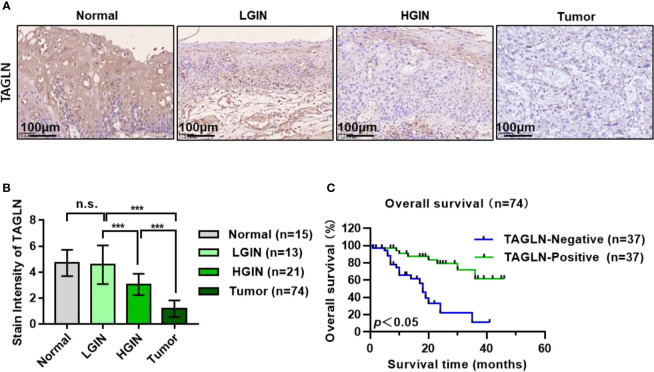
Expression of Transgelin in esophageal squamous cell carcinomas and its relationship with prognosis of patients with esophageal squamous cell carcinomas. **(A)** The expression of Transgelin in Normal, LGIN, HGIN and Tumor tissues was detected by immunohistochemistry. **(B)** Statistical analysis of the expression of Transgelin in Normal, LGIN, HGIN and tumor tissues. The data are presented as the mean ± SEM. ***P < 0.001 by ANOVA analysis. **(C)** Survival analysis of Transgelin expression and prognosis in patients with esophageal squamous cell carcinomas. Kaplan-Meier method was used to calculate the relationship between Transgelin expression and survival prognosis of tumor patients. n.s., no significant.

The expression of Transgelin in different genders, ages, invasion ranges, and clinical stages is shown in **Table 1**. Statistical analysis showed that the expression of Transgelin in ESCC was not significantly correlated with age, gender, and invasion range of patients (P > 0.05), but was significantly correlated with lymph node metastasis, distant metastasis, and clinical stage (**Table 1**, P < 0.05). We further detected the expression changes of Transgelin in normal esophageal tissues, LGIN, HGIN, and tumor by immunohistochemistry (**Figure 6A**). The results showed that the expression of TGLN expression is lower in tumors compared to normal, LGIN and HGIN tissues. The expression level of Transgelin protein in these four tissues showed a continuous declining trend. However, no statistically significant difference was observed in the expression of LGIN in normal esophageal mucosa tissues (**Figure 6B**, P > 0.05). To further clarify the relationship between Transgelin expression and prognosis of ESCC, we followed up the patients using the information collected by the research team. Kaplan–Meier analysis showed that the expression level of Transgelin gene was correlated with the survival prognosis of ESCC patients. Patients with high Transgelin expression had a longer overall survival, whereas those with low Transgelin expression had a poorer prognosis, and the difference was statistically significant (**Figure 6C**, P < 0.05).

### Transgelin Co-Expressed Genes and GO and KEGG Analysis

**Figure 7:**
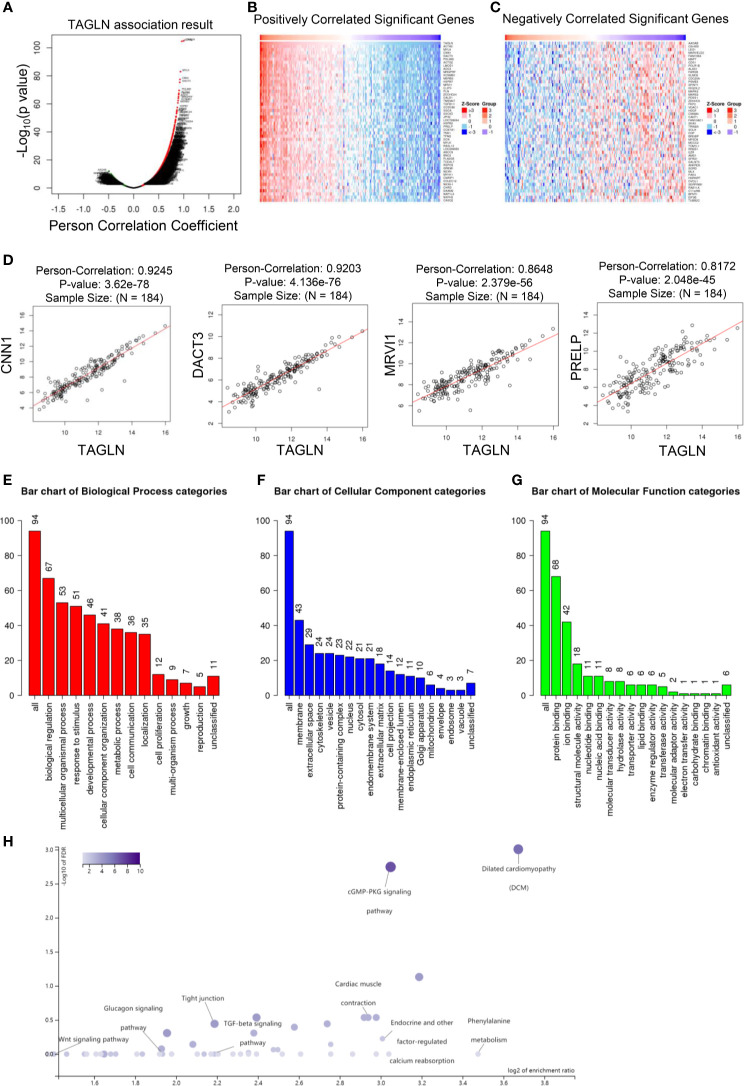
Transgelin co-expressed genes and GO and KEGG enrichment analysis. **(A)** Volcano map analysis results of Transgelin co-expressed genes. **(B)** Heat map analysis of co-expressed genes positively correlated with Transgelin in esophageal squamous cell carcinomas. The red color is the positive gene and the green color is the negative gene. **(C)** Heat map analysis of negatively correlated genes co-expressed with Transgelin in esophageal squamous cell carcinomas. The red color is the positive gene and the green color is the negative gene. **(D)** Correlation analysis of Transgelin, CNN1, DACT3, MRVI1, and PRELP gene expression. The scatter plot showed Pearson correlation between Transgelin expression and other gene expression. **(E)** Results of biological process enrichment analysis of Transgelin co-expressed genes. **(F)** Results of cellular component enrichment analysis of Transgelin co-expressed genes. **(G)** Results of molecular function enrichment analysis of Transgelin co-expressed genes. **(H)** KEGG enrichment analysis results of Transgelin co-expressed genes.

In this study, the public dataset from LinkedOmics database was used to further analyze the expression of Transgelin gene in ESCC and its clinical significance. First, we analyzed the co-expressed genes of Transgelin. **Figure 7A** shows a volcanic map information of Transgelin co-expressed genes. **Figures 7B, C** show heat maps of genes positively and negatively correlated with Transgelin expression, respectively. Through further analysis, we found that Transgelin co-expression was correlated with CNN1, DACT3, MRVI1, and PRELP (**Figure 7D**). The results of Transgelin interaction protein network analysis showed that Transgelin was at the center of the interaction. This finding indicates that Transgelin plays an important role (**Supplementary Figure 1**). Furthermore, we used GO and KEGG analysis functions in LinkedOmics database to enrich Transgelin co-expressed genes. The results of GO enrichment analysis showed that the enriched biological processes mainly included developmental process, metabolic process, cell proliferation, and cell communication. Moreover, the affected molecular functions mainly included protein binding, ion binding, nucleotide binding, transporter activity, and transferase activity (**Figures 7E–G**). In addition, the pathways affected by Transgelin co-expressed genes mainly included the cGMP–PKG signaling pathway, Wnt signaling pathway, and TGF-β signaling pathway (**Figure 7H**).

## Discussion

Early invasion and metastasis of cancer cells are one of the main causes of death in most patients with ESCC (23). Therefore, investigating the invasion and metastasis mechanism of ESCC is important for its treatment and prognosis (24).

Transgelin, also known as SM-22 alpha, is a conserved protein that is mainly expressed in smooth muscles (25, 26). Transgelin expression is decreased in lung and breast cancer (27, 28). Shields et al. (29) showed that the changes of Transgelin might be more sensitive than CEA in the early malignant changes of colorectal mucosa. They found that the decreased expression of Transgelin was also associated with the activation of the Ras oncogene. The latter is activated during the progression from early colorectal adenoma to middle colorectal adenoma. The downregulation of Transgelin expression in tissues may occur as early as in precancerous lesions of the colorectal mucosa. Moreover, this change is associated with the progression and metastasis of colorectal cancer. Some studies have also shown that Transgelin gene expression is upregulated in bladder cancer. It may reflect abnormalities of tumor cells in cell adhesion, cell–cell interaction, migration, and movement. This finding suggests that Transgelin may be closely related to the invasion and metastasis of cancer cells (30). Zhao et al. (31) applied comparative proteomics analysis and found that Transgelin expression was significantly downregulated in colorectal cancer specimens compared with normal colorectal mucosa. Meanwhile, patients with low Transgelin expression levels have poorer prognosis compared with those with high Transgelin expression levels. These studies confirm that the loss of Transgelin gene expression may be an early event in tumor progression.

To investigate the relationship between Transgelin and the invasion and metastasis potential of ESCC, we selected Eca-109 and KYSE-150 cells as experimental cells. The expression of Transgelin in ESCC cells was regulated by siRNA and Transgelin-overexpressing plasmid. After transfection, RT-PCR and Western blot results showed that Transgelin mRNA and protein expressions in Eca-109 and KYSE-150 cells transfected with siRNA were significantly decreased compared with those in the negative control group. However, Transgelin expression was upregulated in cells transfected with Transgelin-overexpressing Transgelin plasmid. The results of *in vitro* proliferation, migration, and invasion experiments showed that the proliferation, migration, and invasion abilities of cells were significantly decreased with the increase of Transgelin expression in Eca-109 and KYSE-150 cells. These results indicated that increased Transgelin expression decreased the malignancy of Eca-109 and KYSE-150 cells. However, Transgelin inhibition enhanced the proliferation, migration, and invasion of ESCC cells. These results indicated that decreased Transgelin expression increased the malignancy of Eca-109 and KYSE-150 cells. We speculate that Transgelin may play a tumor suppressive role in the invasion and metastasis of ESCC.

In this study, the expression of Transgelin in cancer tissues, LGIN, HGIN, and normal esophageal mucosa tissues was further detected by immunohistochemistry. The results showed that the expression of Transgelin protein in ESCC tissues was lower than that in LGIN, HGIN, and normal esophageal mucosa tissues (P < 0.05). This finding suggested that Transgelin may play a role in inhibiting the occurrence of ESCC, and detecting its expression level may provide reference for the early diagnosis of ESCC. Transgelin is associated not only with tumor genesis, but also with tumor invasion and metastasis potential. In this study, the relationship between Transgelin expression and clinical pathological data of patients was analyzed (**Table 1**). The results showed that the expression of Transgelin in ESCC was not significantly correlated with the gender, degree of tumor differentiation, tumor location, and tumor diameter of patients (P > 0.05). However, it was significantly correlated with age, T grade, lymphatic, invasion, and AJCC stage (P < 0.05). This finding suggested that Transgelin may play an important role in the invasion and metastasis of ESCC. The detection of Transgelin expression level in ESCC tissues may have certain reference value for malignant degree evaluation and prognosis judgment of ESCC.

It was found that TAGLN expression was significantly reduced in bladder, breast, and renal cell carcinoma tissues compared with matched normal tissues (32, 33). In this study, Transgelin was found to be less expressed in esophageal squamous cell carcinoma tissues compared with adjacent normal tissues. Studies have shown that Transgelin is regulated by TGF-b/Smad3. The low expression of Transgelin may be caused by the low expression of TGF-β under the effect of esophageal squamous cell carcinoma tumor microenvironment. Fukuchi et al. found that low expression of TGF-β was an adverse prognostic factor in patients with esophageal squamous cell carcinoma (34). This result also supports our hypothesis and conclusion. In addition, activation of Ras-MEK-ERK-MYC signaling pathway can antagonize TGFb and inhibit the expression of Transgelin (29). Ras-MEK-ERK-MYC signaling pathway was highly expressed in esophageal squamous cell carcinoma (35, 36). This may also be the reason for the low expression of Transgelin in esophageal squamous cell carcinoma.

In this study, we found that Transgelin could inhibit the invasion and metastasis of ESCC by regulating the occurrence of EMT in cell experiments. EMT is the transformation of polar epithelial cells into cells that can move freely between the stroma of cells. In this process, cells acquire the ability to infiltrate and migrate (37). EMT is one of the main mechanisms of tumor invasion and metastasis. The disappearance of the polarity of the above skin cells and the acquisition of interstitial characteristics are important features (38). E-cadherin is a commonly used epithelial marker, and Vimentin is a stromal marker. We used qRT-PCR and Western blot to detect the expression of EMT markers in ESCC cells and analyzed the correlation between Transgelin and EMT markers. The results showed that the overexpression of Transgelin in ESCC cells upregulated the expression of E-cadherin. Meanwhile, the expression of Vimentin was inhibited. The results of this study further showed that the downregulation of Transgelin expression was not related to the location of ESCC, but was significantly related to the stage and grade of tumor. The lower the tumor grade, the weaker the expression intensity, which is also similar to the results of previous studies (39, 40). Transgelin may be involved in cell differentiation by binding to actin to stabilize the cytoskeleton. Yang et al. (41) also suggested that Transgelin could inhibit the growth of tumor cells. In breast cancer, Transgelin affects the utilization of IL-8, which in turn affects tumor vascular mimicry (VM). Mechanism studies have shown that Transgelin can regulate IL-8/CXCR2 signaling pathway and regulate the expression of vascular markers. Matrix metalloproteinases (MMPs) are zinc-dependent endopeptidases, which are capable of degrading all kinds of extracellular matrix proteins. Nair RR et al. found that TAGLN was a new inhibitor of MMP9 (42). Li et al. showed that TAGLN could inhibit the proliferation and invasion of colorectal cancer cells by regulating MMP9 and induce their apoptosis (43). In prostate cancer cells, Transgelin inhibits the binding of androgen receptor coactivators to androgen receptors. This in turn inhibits the proliferation and migration of prostate cancer cells (41). In bladder cancer, TAGLN affects cell colony formation, cell migration, and invasion by regulating invadopodia formation and epithelial-mesenchymal transformation (30). In bladder cancer, TAGLN can affect the upregulation of TGF-β-stimulated Slug and MMP14, and thus play a role in regulating EMT (30). In this study, through bioinformatics analysis, it was found that Transgelin had a significant positive co-expression correlation with CNN1 (**Figure 7D**). CNN1 has been reported to inhibit EMT. Liu et al. found that CNN1 regulates the DKK1/Wnt/β−catenin/c−myc signaling pathway by activating TIMP2 to inhibit the invasion, migration and EMT (44).

Esophageal squamous cell carcinoma is still a complex disease of the digestive system. Current treatments are limited and the prognosis is poor. Therefore, it is necessary to conduct further studies on the occurrence, development and metastasis of esophageal squamous cell carcinoma. Transgelin is mainly involved in the remodeling of the actin skeleton. The biological function of Transgelin in tumors is controversial. The role of Transgelin in esophageal squamous cell carcinoma has not been reported. Especially, the expression pattern, function and mechanism of TAGLN in esophageal squamous cell carcinoma are not clear. We found that TAGLN was low expressed in esophageal squamous cell carcinoma and was associated with prognostic characteristics. Transgelin expression was also associated with stage, grade, and overall survival of esophageal squamous cell carcinoma. We found that TAGLN inhibited the proliferation, invasion and migration of esophageal squamous cell carcinoma cells by inhibiting EMT. Our study shows that TAGLN can inhibit the malignant progression of esophageal squamous cell carcinoma. Transgelin may be a promising biomarker and a new target for follow-up therapy.

## Conclusion

Transgelin plays an important role in inhibiting the occurrence, invasion, and metastasis of ESCC. It can provide important reference for early diagnosis, molecular targeted therapy, and prognosis judgment of ESCC. The occurrence of EMT is a complex process, in which multiple genes, proteins, and microenvironment interact together. Transgelin may inhibit the invasion and metastasis of ESCC by regulating EMT.

## Data Availability Statement

The original contributions presented in the study are included in the article/**Supplementary Material**. Further inquiries can be directed to the corresponding authors.

## Ethics Statement

Written informed consent was obtained from the individual(s) for the publication of any potentially identifiable images or data included in this article.

## Author Contributions

WZ and BW designed the study. BY, WZ, QC, CW, SS, LZ, and ZZ performed the experiments and analyzed the data. BY, QC, and WZ performed the data analysis. BY, QC, and WZ wrote the initial draft of the paper, with contributions from all authors. All authors contributed to the article and approved the submitted version.

## Conflict of Interest

The authors declare that the research was conducted in the absence of any commercial or financial relationships that could be construed as a potential conflict of interest..

## Publisher’s Note

All claims expressed in this article are solely those of the authors and do not necessarily represent those of their affiliated organizations, or those of the publisher, the editors and the reviewers. Any product that may be evaluated in this article, or claim that may be made by its manufacturer, is not guaranteed or endorsed by the publisher.

## Supplementary Material

Supplementary Figure 1 Prognostic analysis of Transgelin in breast cancer, liver hepatocellular carcinoma and sarcoma.

Supplementary Figure 2 PPI interaction network analysis of Transgelin interacting proteins.

Supplementary Figure 3 Inhibition of Transgelin promoted the proliferation, migration and invasive abilities of esophageal squamous cell carcinomas cells *in vitro*. **(A)** Eca-109 and KYSE-150 cells transfected with siRNA-2 (Transgelin: 5’-GCUGAAGAAUGGCGUGAUUTT-3’, Genepharma) to silence Transgelin, respectively. The expression of Transgelin was verified by qRT-PCR. **(B)** Eca-109 and KYSE-150 cells transfected with siRNA to silence Transgelin, respectively. The expression of Transgelin was verified by western blotting. GAPDH and β-Actin were used as internal controls. **(C)** The effects of knockdown of Transgelin on the proliferation ability was analyzed by CCK-8 kit in Eca-109 cells. **(D)** The effects of knockdown of Transgelin on the proliferation ability was analyzed by CCK-8 kit in KYSE-150 cells. **(E)** The effects of knockdown of Transgelin on the migration ability was analyzed by Scratch test in Eca-109 cells. **(F)** The effects of knockdown of Transgelin on the migration ability was analyzed by Scratch test in KYSE-150 cells. **(G)** The effects of knockdown of Transgelin on the migration ability was analyzed by Transwell assay in Eca-109 and KYSE-150 cells. **(H)** The effects of knockdown of Transgelin on the invasion ability was analyzed by Transwell assay in Eca-109 and KYSE-150 cells. The data are presented as the mean ± SEM of three independent experiments. **P* < 0.05, ***P* < 0.01, and ****P* < 0.001 by two-tailed Student’s t-test.

Supplementary Figure 4 Transgelin knockdown promoted EMT of ESCC. The protein levels of E-cadherin, Claudin-1, N-cadherin, γ-Catenin and Vimentin after Transgelin knockdown were detected by Western blot in Eca-109 **(A)** and KYSE-150 **(B)** cell lines.

## Abbreviations

EMT, epithelial-to-mesenchymal transition; IHC, immunohistochemistry; TAGLN, transgelin; GO, Gene Ontology; KEGG, Kyoto Encyclopedia of Genes and Genomes.
