# Corrigendum: Transgelin Inhibits the Malignant Progression of Esophageal Squamous Cell Carcinomas by Regulating Epithelial–Mesenchymal Transition

**DOI:** 10.3389/fonc.2022.888600

**Published:** 2022-03-29

**Authors:** Boli Yang, Qiuyu Chen, Changshan Wan, Siyuan Sun, Lanping Zhu, Zhizhong Zhao, Weilong Zhong, Bangmao Wang

**Affiliations:** ^1^ Department of Gastroenterology and Hepatology, Tianjin Medical University General Hospital, Tianjin Institute of Digestive Disease, Tianjin, China; ^2^ Department of Digestive Diseases, Jincheng General Hospital, Shanxi, China

**Keywords:** esophageal squamous cell carcinoma (ESCC), transgelin, epithelial mesenchymal transition (EMT), invasion, metastasis, proliferation

An author name was incorrectly spelled as “Bangmang Wang”. The correct spelling is “Bangmao Wang”.

In the published article, there was an error in Affiliation 1. Instead of “Department of Gastroenterology and Hepatology, General Hospital, Tianjin Medical University, Tianjin Institute of Digestive Disease, Tianjin, China”, it should be “Department of Gastroenterology and Hepatology, Tianjin Medical University General Hospital, Tianjin Institute of Digestive Disease, Tianjin, China”. The authors apologize for this error and state that this does not change the scientific conclusions of the article in any way. The original article has been updated.

In the published article, there was an error in Affiliation 2. Instead of “Department of Digestive Diseases, General Hospital of Jincheng, Tianjin, China”, it should be “Department of Digestive Diseases, Jincheng General Hospital, Shanxi, China”.

The authors apologize for these errors and state that they do not change the scientific conclusions of the article in any way. The original article has been updated.

## Publisher’s Note

All claims expressed in this article are solely those of the authors and do not necessarily represent those of their affiliated organizations, or those of the publisher, the editors and the reviewers. Any product that may be evaluated in this article, or claim that may be made by its manufacturer, is not guaranteed or endorsed by the publisher.

