# Supplementary figures and images for: Transgelin Inhibits the Malignant Progression of Esophageal Squamous Cell Carcinomas by Regulating Epithelial–Mesenchymal Transition
Source: Front Oncol. 2021 Aug 26;11:709486. doi: 10.3389/fonc.2021.709486 (PMC8450671; doi:10.3389/fonc.2021.709486)

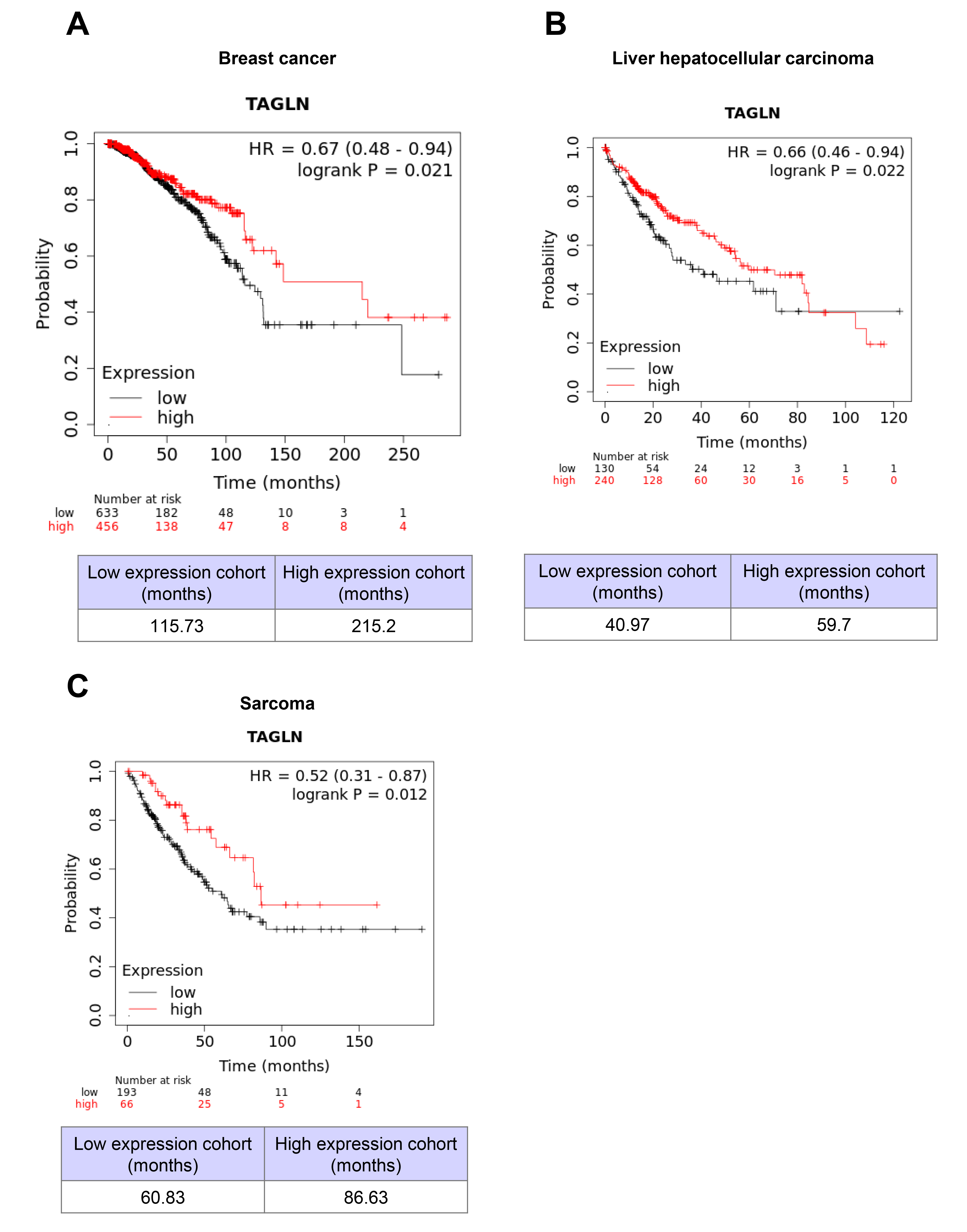

Supplement: Supplementary Figure 1 — Prognostic analysis of Transgelin in breast cancer, liver hepatocellular carcinoma and sarcoma. [file Image_1.jpeg]

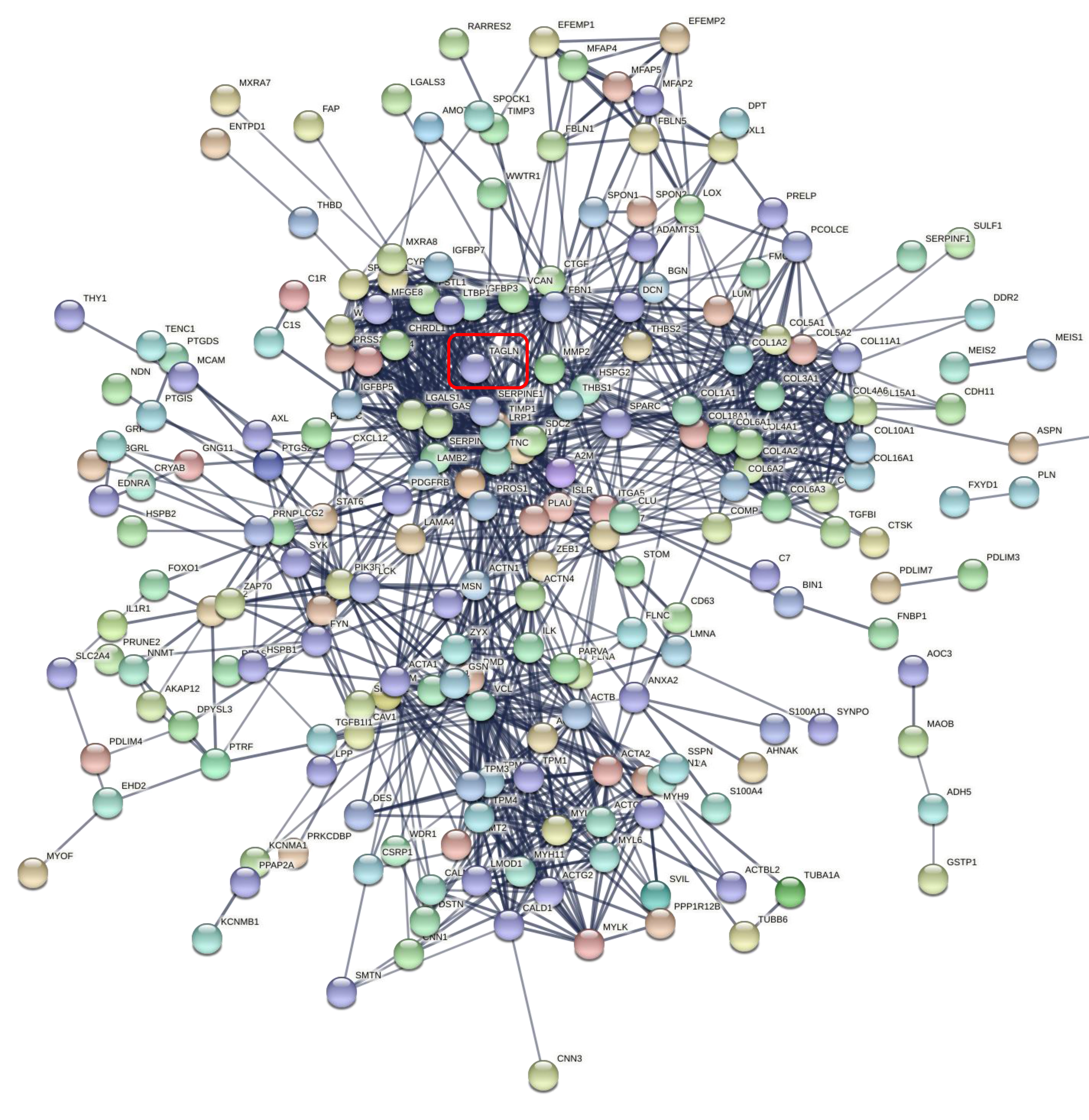

Supplement: Supplementary Figure 2 — PPI interaction network analysis of Transgelin interacting proteins. [file Image_2.jpeg]

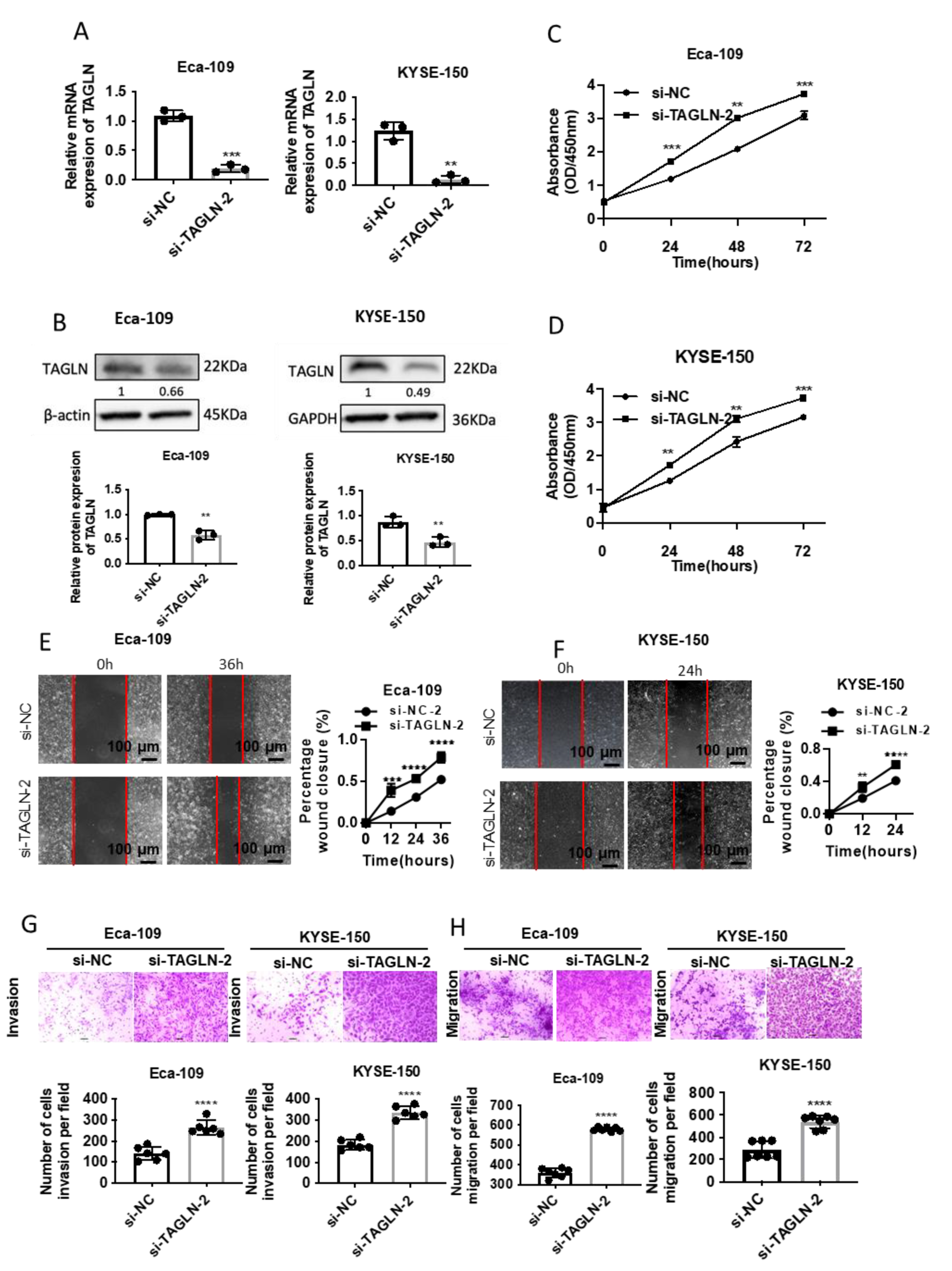

Supplement: Supplementary Figure 3 — Inhibition of Transgelin promoted the proliferation, migration and invasive abilities of esophageal squamous cell carcinomas cells in vitro. (A) Eca-109 and KYSE-150 cells transfected with siRNA-2 (Transgelin: 5’-GCUGAAGAAUGGCGUGAUUTT-3’, Genepharma) to silence Transgelin, respectively. The expression of Transgelin was verified by qRT-PCR. (B) Eca-109 and KYSE-150 cells transfected with siRNA to silence Transgelin, respectively. The expression of Transgelin was verified by western blotting. GAPDH and β-Actin were used as internal controls. (C) The effects of knockdown of Transgelin on the proliferation ability was analyzed by CCK-8 kit in Eca-109 cells. (D) The effects of knockdown of Transgelin on the proliferation ability was analyzed by CCK-8 kit in KYSE-150 cells. (E) The effects of knockdown of Transgelin on the migration ability was analyzed by Scratch test in Eca-109 cells. (F) The effects of knockdown of Transgelin on the migration ability was analyzed by Scratch test in KYSE-150 cells. (G) The effects of knockdown of Transgelin on the migration ability was analyzed by Transwell assay in Eca-109 and KYSE-150 cells. (H) The effects of knockdown of Transgelin on the invasion ability was analyzed by Transwell assay in Eca-109 and KYSE-150 cells. The data are presented as the mean ± SEM of three independent experiments. *P < 0.05, **P < 0.01, and ***P < 0.001 by two-tailed Student’s t-test. [file Image_3.jpg]

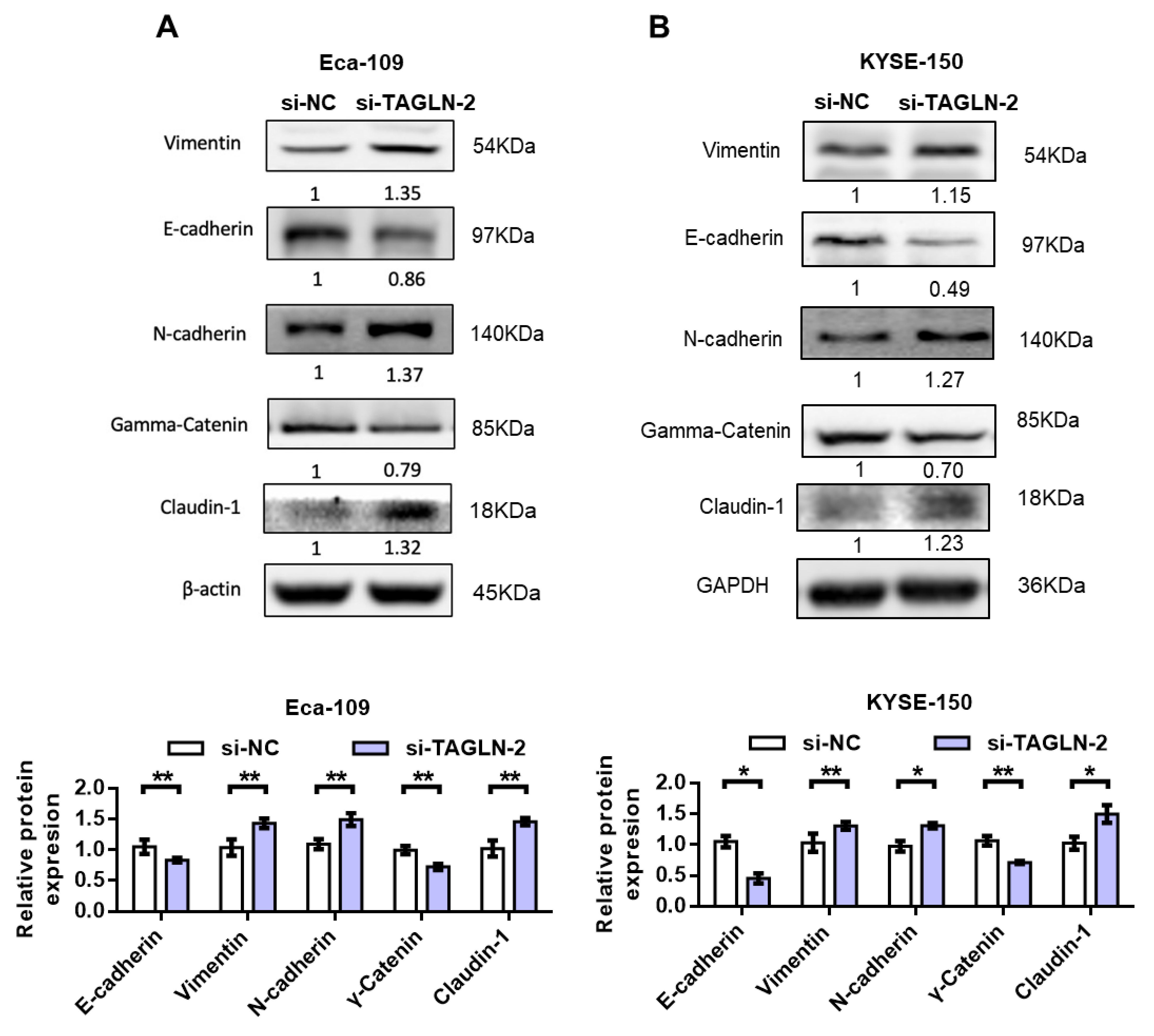

Supplement: Supplementary Figure 4 — Transgelin knockdown promoted EMT of ESCC. The protein levels of E-cadherin, Claudin-1, N-cadherin, γ-Catenin and Vimentin after Transgelin knockdown were detected by Western blot in Eca-109 (A) and KYSE-150 (B) cell lines. [file Image_4.jpeg]
